# Studies on the analysis of 25-hydroxyvitamin D_3_ by isotope-dilution liquid chromatography–tandem mass spectrometry using enzyme-assisted derivatisation

**DOI:** 10.1016/j.bbrc.2014.01.088

**Published:** 2014-04-11

**Authors:** Jonas Abdel-Khalik, Peter J. Crick, Graham D. Carter, Hugh L. Makin, Yuqin Wang, William J. Griffiths

**Affiliations:** a Institute of Mass Spectrometry, College of Medicine, Swansea University, Singleton Park, Swansea SA2 8PP, UK; b DEQAS, Imperial College Healthcare NHS Trust, Clinical Biochemistry Department, Charing Cross Hospital, Fulham Palace Rd, London W6 8RF, UK; c Barts and the Royal London School of Medicine and Dentistry, Queen Mary University of London, London E1 2AD, UK

**Keywords:** 1α,25-(OH)_2_D_3_, 1α,25-dihydroxyvitamin D_3_, 3-epi-25-OHD_3_, 3-epi-25-hydroxyvitamin D_3_, 17βHSD10, 17β-hydroxysteroid dehydrogenase type 10, 24,25-(OH)_2_D_3_, 24,25-dihydroxyvitamin D_3_, 25-OHD_2_, 25-hydroxyvitamin D_2_, 25-OHD_3_, 25-hydroxyvitamin D_3_, [^2^H_6_]25-OHD_3_, 25-[26,26,26,27,27,27-^2^H_6_]hydroxyvitamin D_3_, β-NAD^+^, β-nicotinamide adenine dinucleotide, CYP, cytochrome P450, CV, coefficient of variation, GP, Girard P reagent, DEQAS, Vitamin D External Quality Assessment Scheme, LC, liquid chromatography, MS, mass spectrometry, MS^n^, mass spectrometry with multistage fragmentation, NIST, National Institute of Standards and Technology, Py, pyridine, RIC, reconstructed ion chromatogram, SPE, solid phase extraction, SRM, standard reference material, UV, ultra violet, Vitamins D, Serum, Cholesterol oxidase, 17βHSD10, Girard P reagent, LC–MS^n^

## Abstract

The total serum concentration of 25-hydroxyvitamins D (25-hydroxyvitamin D_3_ and 25-hydroxyvitamin D_2_) is currently used as an indicator of vitamins D status. Vitamins D insufficiency is claimed to be associated with multiple diseases, thus accurate and precise reference methods for the quantification of 25-hydroxyvitamins D are needed. Here we present a novel enzyme-assisted derivatisation method for the analysis of vitamins D metabolites in adult serum utilising 25-[26,26,26,27,27,27-^2^H_6_]hydroxyvitamin D_3_ as the internal standard. Extraction of 25-hydroxyvitamins D from serum is performed with acetonitrile, which is shown to be more efficient than ethanol. Cholesterol oxidase is used to oxidize the 3β-hydroxy group in the vitamins D metabolites followed by derivatisation of the newly formed 3-oxo group with Girard P reagent. 17β-Hydroxysteroid dehydrogenase type 10 is shown to oxidize selectively the 3α-hydroxy group in the 3α-hydroxy epimer of 25-hydroxyvitamin D_3_. Quantification is achieved by isotope-dilution liquid chromatography–tandem mass spectrometry. Recovery experiments for 25-hydroxyvitamin D_3_ performed on adult human serum give recovery of 102–106%. Furthermore in addition to 25-hydroxyvitamin D_3_, 24,25-dihydroxyvitamin D_3_ and other uncharacterised dihydroxy metabolites, were detected in adult human serum.

## Highlights

• New method for the analysis of 25-hydroxyvitamin D_3_ exploiting Girard P derivatisation.
• Method also applicable to vitamin D_3_, 1α,25- and 24,25-dihydroxyvitamin D_3_.
• By modification of the method 3-epi-25-hydroxyvitamin D_3_ can also be analysed.

## 1 Introduction

**Fig. 1:**
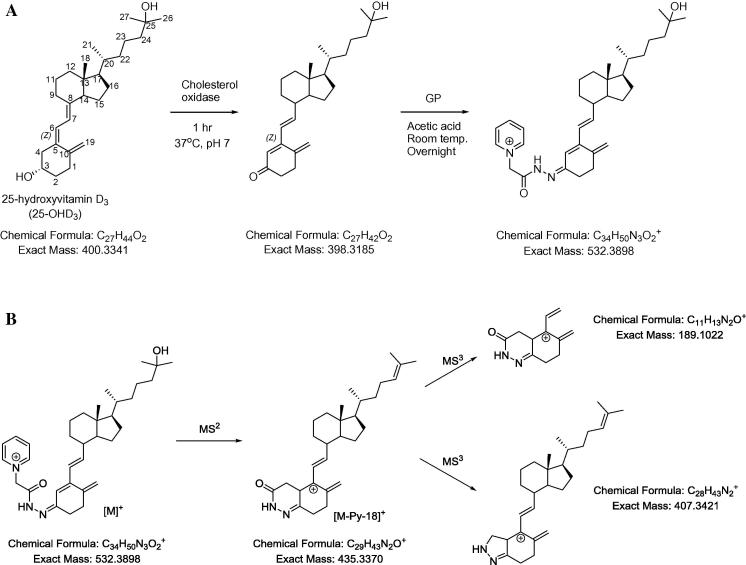
(A) Overview of the enzyme-assisted derivatisation method for 25-OHD_3_. (B) MS^2^ and MS^3^ fragmentation pathways for 25-OHD_3_.

Vitamin D_2_ and D_3_ belong to the class of *seco*steroids which, compared to common steroids are characterised by having an open B ring between carbons 9 and 10 [1]. As in oxysterols the configuration around the C-5–C-6 double bond is Z (Fig. 1A). Ultra violet (UV) B light from the sun causes the formation of vitamin D_3_ from 7-dehydrocholesterol in the upper dermis layers of the skin [1–5]. Vitamin D_2_ is formed in plants and fungi following photolysis of ergosterol by UV irradiation and appears in dietary supplements and fortified foods [1,4]. Vitamins D_3_ and D_2_ are metabolized in the liver by cytochochrome P450 (CYP) enzymes to 25-hydroxyvitamin D_3_ (25-OHD_3_) and D_2_ (25-OHD_2_), respectively. 25-Hydroxyvitamins D are further metabolized, primarily in the kidney, but also in other target tissues to their active metabolites 1α,25-dihydroxyvitamin D_3_ (1α,25-(OH)_2_D_3_) and D_2_ (1α,25-(OH)_2_D_2_) and the metabolites 24,25-dihydroxyvitamin D_3_ (24,25-(OH)_2_D_3_) and D_2_ (24,25-(OH)_2_D_2_) [1].

Insufficiency in vitamins D is claimed to be associated with diseases such as cardiovascular disease, hypertension, diabetes, cancer, skin disorders and autoimmune disease [2,3,6]. Due to the recent increase in numbers of vitamins D analysis performed worldwide there is a need for accepted reference methods for determination of 25-OHD_2_ and 25-OHD_3_ in human serum, the indicators of vitamins D status [4]. Liquid chromatography (LC) offers separation of 25-OHD_2_ and 25-OHD_3_ from each other and from much matrix interference, including isobaric interferences from e.g. the endogenous 3α-hydroxy epimer of 25-OHD_3_ (3-epi-25-OHD_3_) [7,8]. Use of isotope dilution mass spectrometry (MS) typically further limits matrix effects and improves precision and accuracy [8]. LC–MS has been proposed as the “gold standard” for quantification of 25-hydroxyvitamins D in serum [8]. Within the last ten years LC–MS has grown in use and today about 11% of clinical laboratories use LC–MS for vitamins D analysis [7,8]. Currently there are two procedures based on LC–MS which have been accepted as reference method procedures by the Joint Committee for Traceability in Laboratory Medicine [9,10].

The aim of this report is to introduce a novel procedure for quantification of 25-OHD_3_ in adult human serum, which involves enzyme-assisted derivatisation with cholesterol oxidase and derivatisation with Girard P (GP) reagent. As vitamin D_2_ is used pharmaceutically to treat vitamins D deficiency mostly in the USA, and the adult human serum analysed in this study originated from the UK, 25-OHD_2_ was not included in our studies [2]. The new method offers improved sensitivity and most importantly specificity for vitamins D analysis compared to the two current reference methods [9,10]. Briefly the method involves oxidation of the 3β-hydroxy group of 25-OHD_3_ with cholesterol oxidase followed by derivatisation of the newly formed 3-oxo group with GP reagent (Fig. 1A). The method is a modification of the published protocol for oxysterol analysis [11,12].

## 2 Materials and methods

25-OHD_3_ was from Standard Reference Material (SRM) 2972 from the National Institute of Standards and Technology (NIST, Gaithersburg, Maryland, USA). It was dissolved in ethanol at a certified concentration of 846.0 nmol/L (338.9 ng/mL). 3-Epi-25-OHD_3_ (⩾98% chemical purity) in ethanol at 100 μg/mL was purchased from Sigma–Aldrich (Dorset, UK). 1α,25-(OH)_2_D_3_ and 24,25-(OH)_2_D_3_ were from Sigma–Aldrich. The internal standard 25-[26,26,26,27,27,27-^2^H_6_]hydroxyvitamin D_3_ ([^2^H_6_]25-OHD_3_) was from Medical Isotopes, Inc. (Pelham, NH, USA) and was of chemical and isotopic purity >99% and >98%, respectively. Cholesterol oxidase from *Streptomyces sp*, glutathione S-transferase tagged human 17β-hydroxysteroid dehydrogenase 10 (17βHSD10) and β-nicotinamide adenine dinucleotide (β-NAD^+^) hydrate were from Sigma–Aldrich (Dorset, UK). GP reagent [1-(carboxymethyl)pyridinium chloride hydrazide] was purchased from TCI Europe (Oxford, UK). [^2^H_5_]GP reagent was synthesised in house. Solid phase extraction (SPE) cartridges, Certified Sep-Pak C18, 200 mg (3 cm^3^), and 60 mg Oasis HLB (3 cm^3^), were from Waters Inc. (Elstree, UK). Solvents were obtained from Fisher-Scientific (Loughborough, UK). Acetic acid and formic acid were of AnalaR NORMAPUR grade (BDH, VWR, Lutterworth, UK). Potassium dihydrogen phosphate and potassium pyrophosphate decahydrate were from Sigma–Aldrich.

Stock solutions of 1α,25-(OH)_2_D_3_ and 24,25-(OH)_2_D_3_ were prepared by dissolving 100 μg in 1 mL of absolute ethanol (100 ng/μL). A stock solution of [^2^H_6_]25-OHD_3_ was prepared by dissolving 1.70 mg in 17 mL absolute ethanol (100 ng/μL). All stock solutions were stored in the dark. Working solutions (1 ng/μL) were made immediately before sample preparation by diluting 10 μL stock solution in 990 μL of absolute ethanol.

### 2.1 Procedure

#### 2.1.1 Sample preparation

Sample preparation of serum was largely performed as previously described [12]. In brief, 100 μL of serum was added drop-wise to a solution of acetonitrile (1.05 mL) containing 1 ng of [^2^H_6_]25-OHD_3_. After 10 min sonication in an ultrasonic bath the solution was centrifuged at 14,000*g* at 4 °C for 30 min. The supernatant was dried under vacuum using a ScanLaf ScanSpeed vacuum concentrator and reconstituted in 1.05 mL of absolute ethanol and sonicated for 15 min. Water (0.45 mL) was added dropwise and ultrasonication continued for a further 5 min. The final sample solution of 1.5 mL 70% ethanol was loaded onto a 200 mg Certified Sep-Pak C18 cartridge pre-conditioned with 4 mL of absolute ethanol and with 6 mL of 70% ethanol. The solvent flow through the column was at a rate of ∼0.25 mL/min assisted by negative pressure at the column outlet generated by a vacuum manifold (Agilent Technologies). The flow-through (1.5 mL) was combined with a column wash of 70% ethanol (5.5 mL) to give fraction SPE1-Fr1 (7 mL). A second fraction (SPE1-Fr2) was collected by eluting with a further 4 mL of 70% ethanol before fraction 3 containing cholesterol was eluted with 2 mL of absolute ethanol (SPE1-Fr3). Finally, a fourth fraction eluted with a second 2 mL of absolute ethanol, (SPE1-Fr4). Each fraction was divided into two equal fractions (A) and (B) and allowed to dry overnight under reduced pressure. Lyophilised material was reconstituted in 100 μL of propanol-2-ol. The remainder of the procedure, oxidation with cholesterol oxidase and GP derivatisation followed by SPE purification, was performed as previously described with the exception that Sep-Pak C18 cartridges were replaced by Oasis HLB cartridges [11–13].

#### 2.1.2 LC–MS and MS^n^ analysis

Analysis was performed on a LTQ-Orbitrap Velos (Thermo Fisher Scientific, UK) equipped with an electrospray probe, and a Dionex Ultimate 3000 LC system (Dionex, UK), essentially as described by Griffiths et al. [12]. The only major difference was in the MS^3^ events where in the current study we exploited the neutral losses of 97.05 Da ([M]^+^ → [M-Py-18]^+^→) rather than 79.04 Da ([M]^+^ → [M-Py]^+^→) as 25-hydroxylated metabolites of vitamins D lose water in addition to pyridine in the initial fragmentation event (Fig. 1B) while oxysterols mostly lose pyridine [12].

#### 2.1.3 Quantification

Serum 25-OHD_3_ was quantified by stable isotope dilution LC–MS against [^2^H_6_]25-OHD_3_ reference standard.

### 2.2 Optimisation of extraction

Acetonitrile and ethanol were compared in their ability to extract 25-OHD_3_ in serum. Performance of a single-step extraction was compared against a two-step extraction i.e., re-extraction of the pellet following the initial extraction. Extraction in acetonitrile was performed as stated above while extraction in ethanol was performed as described by Griffiths et al. [11,12]. The supernatant generated by the second extraction was either combined with that from the first extraction or processed separately.

### 2.3 Recovery experiments

#### 2.3.1 Standard addition of [^2^H_6_]25-OHD_3_

Known amounts of [^2^H_6_]25-OHD_3_ (2, 4 or 6 ng) were added to 100 μL of serum (batch DEQAS423, the endogenous level of 25-OHD_3_ was predetermined using 1 ng of internal standard), and extracted once using acetonitrile as described above. Each experiment was performed in triplicate. Recovery was determined at each concentration of added internal standard by dividing the experimentally measured concentration ratio of 25-OHD_3_ to [^2^H_6_]25-OHD_3_ with the theoretical concentration ratio (Eq. (1)) [9].

(1) $$\%\;\text{Recovery}=\left\{{\left(\left[25\text{-}{\text{OHD}}_{3}\right]/[{}^{2}{\text{H}}_{6}]25\text{-}{\text{OHD}}_{3}\right)}_{\text{exp}}/{\left([25\text{-}{\text{OHD}}_{3}]/[{}^{2}{\text{H}}_{6}]25\text{-}{\text{OHD}}_{3}\right)}_{\text{theor}}\right\}\times 100\%$$

#### 2.3.2 Standard addition of 25-OHD_3_

A second recovery experiment was performed by adding known amounts of 25-OHD_3_ (1, 2, 4 or 6 ng) to 100 μL serum (batch DEQAS424, the endogenous level of 25-OHD_3_ was predetermined), and extracting once using acetonitrile. The internal standard [^2^H_6_]25-OHD_3_ (1 ng) was added to each sample. Each experiment was performed in triplicate. Recovery was determined at each concentration of added 25-OHD_3_ by dividing the experimentally measured serum concentration of 25-OHD_3_ with the theoretical concentration (Eq. (2)) [9].

(2) $$\%\;\text{Recovery}=\left\{{\left[25\text{-}{\text{OHD}}_{3}\right]}_{\text{exp}}/{\left[25\text{-}{\text{OHD}}_{3}\right]}_{\text{theor}}\right\}\times 100\%$$

### 2.4 Selective derivatisation of 3-epi-25-OHD_3_

Oxidation of 400 ng of 3-epi-25-OHD_3_ or 25-OHD_3_ by 17βHSD10 was essentially performed as described in the literature for steroid hormones and bile acids [14,15]. 30 mg of β-NAD^+^ was dissolved in 1 mL of 100 mM pyrophosphate buffer pH 8.9. 3-Epi-25-OHD_3_ or 25-OHD_3_ dissolved in ethanol was added (10 μL), giving a final concentration of 1% ethanol. Finally 1 μg of 17βHSD10 was added, giving an enzyme concentration of 1 μg/mL. After incubation at room temperature for 24 h, 40 μL of methanol was added (content of organic solvent is now 5%). The mixture, followed by a 0.5 mL rinse (5% methanol) of the reaction tube, was loaded on a 60 mg HLB cartridge previously washed with 6 mL of methanol and conditioned 6 mL of 5% methanol. The loaded cartridge was washed with 6 mL of 5% methanol. Elution of *seco*sterols was performed with 2 mL of methanol into which 1 mL of water was added to make the solution 67% methanol and quenching any residual enzyme activity [11–13]. Glacial acetic acid (150 μL) was then added followed by GP reagent (150 mg) and the mixture left at room temperature overnight. Finally SPE recycling on a 60 mg Oasis HLB cartridges to remove excess derivatisation reagent was carried out as described previously [11–13]. LC–MS^n^ analysis was performed as described above (Section 2.1.2).

## 3 Results and discussion

### 3.1 General considerations related to the methodology

**Fig. 2:**
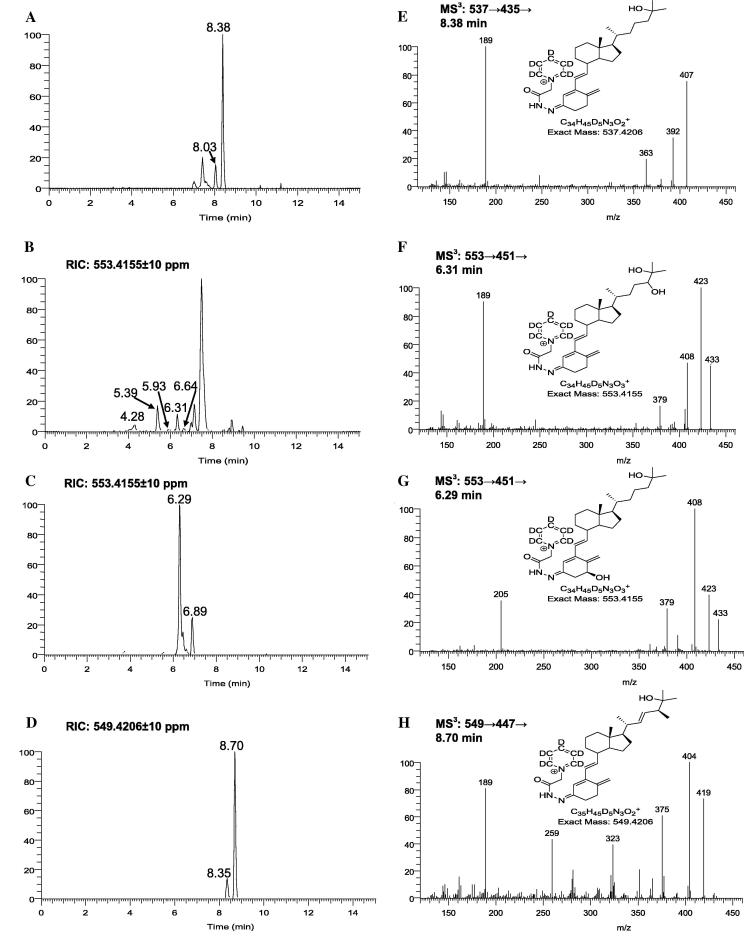
LC–MS RIC ± 10 ppm appropriate to GP-derivatised (A) monohydroxyvitamin D_3_, (B) dihydroxyvitamin D_3_, authentic (C) 1,25-(OH)_2_D_3_ and (D) 25-OHD_2_. RIC shown in (A) and (B) are for adult human serum, (C) and (D) are of authentic standards (25 pg on-column). GP-derivatives form *syn* and *anti* conformers about C-3 this is evident in (A) where 25-OHD_3_ elutes in two peaks at 8.03 and 8.38 min, in (B) where 24,25-(OH)_2_D_3_ elutes at 5.93 and 6.31 min and in (D) where 25-OHD_2_ elutes at 8.35 and 8.70 min. Shown in (E–H) are MS^3^ ([M]^+^ → [M-Py-18]^+^→) spectra of GP-derivatised 25-OHD_3_, 24,25-(OH)_2_D_3_, 1α,25-(OH)_2_D_3_ and 25-OHD_2_. In this example [^2^H_5_]GP reagent was used. Due to the presence of the 1α-hydroxy group in 1α,25-(OH)_2_D_3_ its major fragment in the low *m*/*z* range is *m*/*z* 205 rather than *m*/*z* 189 as seen for 25-OHD_3_, 24,25-(OH)_2_D_3_ and 25-OHD_2_. This agrees with the fragmentation pathway presented in Fig. 1B. The major unannotated peak in (B) corresponds to GP-derivatised 3β-hydroycholest-(25R)-5-en-26-oic acid, a major component of human serum [12].

GP derivatisation of 25-OHD_3_ provides multiple advantages including increased solubility in mobile phases commonly used for LC–MS, enhanced ionisation, characteristic fragmentation patterns in MS^2^ i.e. loss of pyridine (Py) and water giving [M-Py-18]^+^ ions (Fig. 1B), and structurally informative MS^3^ spectra ([M]^+^ → [M-Py-18]^+^→) with particularly intense fragment ions at *m*/*z* 189 for 25-OHD_3_ (Fig. 1B) and its side-chain oxidised metabolites (Fig. 2E, F, H) or *m*/*z* 205 for 1,25-(OH)_2_D_3_ and its metabolites (Fig. 2G). The current method, with specific fragment ions at *m*/*z* 189 and 205, provides advantages over other LC–MS/MS procedures based on the loss of water or other nonspecific fragmentations [9]. With respect to sensitivity, LC–MS analysis of GP-derivatised 25-OHD_3_ in serum (16.54 ng/mL, on-column injection of 6.8 pg) gives a signal-to-noise ratio of approximately 60 (Fig. 2A). In comparison the limits of detection are 10 and 40 pg on-column for the two current LC–MS reference methods [9,10]. By generating reconstructed-ion chromatograms (RIC) for the transitions [M]^+^ → [M-Py-18]^+^ → *m*/z 189 or 205 appropriate for dihydroxy metabolites of vitamin D_3_, the presence of possible vitamin D_3_ metabolites eluting at 4.28, 5.39, 5.93, 6.31, and 6.64 min was revealed (Fig. 2B). Analysis of an authentic standard of 24,25-(OH)_2_D_3_ confirmed the peaks at 5.93 and 6.31 min to be the *syn*/*anti* conformers of 24,25-(OH)_2_D_3_.

Although the derivatisation protocol exploited here was originally developed for the analysis of oxysterols [11,12], we now show that it is equally applicable to vitamins D metabolites. In Fig. 2 we show the utility of the method to the analysis of the *seco*sterols, 25-OHD_3_ and 24,25-(OH)_2_D_3_ in adult human serum and to 1,25-(OH)_2_D_3_ and 25-OHD_2_ standards. Thus, an open B ring in *seco*sterols does not prevent their oxidation by cholesterol oxidase.

As 3-epi-25-OHD_3_ and 25-OHD_3_ only differ in the configuration of the 3-hydroxy group, following oxidation with cholesterol oxidase then GP derivatisation their products are identical. Therefore 3-epi-25-OHD_3_ could interfere with the analysis of 25-OHD_3_. However, we find that in 1 h incubations with cholesterol oxidase the efficiency of oxidation of 3-epi-25-OHD_3_ is less than 10% that of 25-OHD_3_. As the adult serum concentration of 3-epi-25OHD_3_ is typically only 4% that of 25-OHD_3_ [8], interference by 3-epi-25-OHD_3_ will result in <1% overestimation of the serum concentration of 25-OHD_3_. However, in cases where the presence of 3-epi-25OHD_3_ is suspected (infants <1 year) its exact level can be determined using the enzyme 17βHSD10 (see Section 3.2 below), and by incorporating the internal standard 3-epi-25-[6,19,19-^2^H_3_]OHD_3_ its relative contribution to the peak of GP derivatised 25-OHD_3_ can be determined.

### 3.2 Novel derivatisation of 3-epi-25-OHD_3_

**Fig. 3:**
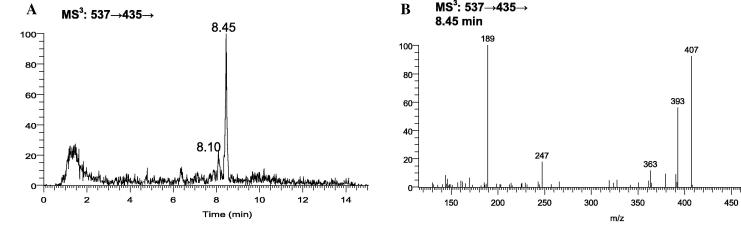
(A) MS^3^ ([M^+^] → [M-Py-18]^+^→) TIC of 3-epi-25-OHD_3_ oxidized with 17βHSD10 and derivatised with GP reagent. (B) MS^3^ spectrum of 3-epi-25-OHD_3_-GP eluting at 8.45 min. As in Fig. 2 [^2^H_5_]GP reagent was used.

17βHSD10 is a multifunctional enzyme capable of oxidizing multiple steroids with 3α-, 7α-, 7β-, 17β-, 20β-, or 21-hydroxy group if its cofactor, β-NAD^+^ is present [14–16]. Thus, 17βHSD10 could be an enzyme capable of oxidizing 3-epi-25-OHD_3_ (3α-hydroxy) but not 25-OHD_3_ (3β-hydroxy), thereby allowing selective analysis of 3-epi-25-OHD_3_ following GP derivatisation. The biological significance if any of 3-epi-25-OHD_3_ remains to be elucidated, but it contributes 9–61% of the total 25-hydroxyvitamins D in infant (<1 year) sera [8]. Oxidation of authentic standards of 3-epi-25-OHD_3_ and 25-OHD_3_ by 17βHSD10 followed by GP derivatisation proved that the enzyme was capable of oxidizing 3-epi-25-OHD_3_ (Fig. 3A and B), but no product of 25-OHD_3_ oxidation was observed. Currently analytical methods in the literature rely on chromatographic resolution of 3-epi-25-OHD_3_ from 25-OHD_3_ [8]. Use of 17βHSD10 to oxidize selectively 3-epi-25-OHD_3_ provides an alternative route for its specific analysis.

### 3.3 Optimisation of extraction

In Supplementary Table S1 a comparison of the amounts of 25-OHD_3_ recovered from adult serum using different extraction methods is given. Extraction with ethanol was less efficient than with acetonitrile. Two-step extraction with acetonitrile was no more efficient than a single extraction. The concentration of 25-OHD_3_ determined using the one-step acetonitrile extraction was 17.76 ± 0.79 ng/mL (mean ± SD), with a coefficient of variation (CV) of <5%. This agrees well with the value of 18.07 ng/mL certified by NIST for the sample using their LC–MS reference method [9].

### 3.4 Recovery experiments

Supplementary Tables S2 and S3 show recovery data from experiments exploiting standard addition of [^2^H_6_]25-OHD_3_ (102.0–106.3%) and of 25-OHD_3_ (101.2–104.9%), respectively, to adult human serum. The within-batch precision was <6% in both experiments.

In conclusion we report an LC–MS^n^ method based on enzyme-assisted derivatisation for the analysis of vitamins D metabolites, including 3-epi-25-OHD_3_. In addition to being accurate and robust in quantifying the serum level of adult 25-OHD_3_, diagnostic MS^3^ fragment ions of *m*/*z* 189 and 205 confirm the identification of vitamins D metabolites.
