# Supplementary material for: Studies on the analysis of 25-hydroxyvitamin D3 by isotope-dilution liquid chromatography–tandem mass spectrometry using enzyme-assisted derivatisation
Source: Biochem Biophys Res Commun. 2014 Apr 11;446(3):745–50. doi: 10.1016/j.bbrc.2014.01.088 (PMC4000436; doi:10.1016/j.bbrc.2014.01.088)
Supplement: Supplementary data 1 — Supplementary Tables. [file mmc1.docx]

**Table S1. Extraction of 25-OHD_3_ from adult serum using different solvents.**

| Extraction | Mean concentration ± SD (ng/mL) | CV  (%) |
| --- | --- | --- |
| *1xMeCN^a^* | 17.8±0.8  (n=3) | 4.5 |
|  |  |  |
|  |  |  |
| *2^nd^ MeCN^b^* | NA  (n=2) | NA |
|  |  |  |
| *2xMeCN^c^* | 17.9±1.3  (n=3) | 7.3 |
|  |  |  |
|  |  |  |
| *1xEtOH^a^* | 14.2±0.8  (n=2) | 5.9 |
|  |  |  |
| *2^nd^ EtOH^b^* | NA  (n=2) | NA |
|  |  |  |
| *2xEtOH^c^* | 14.6±1.4  (n=2) | 9.3 |
|  |  |  |

Abbreviations: MeCN, acetonitrile; EtOH, ethanol; NA, not applicable.

Serum used batch DEQAS435. Certified serum concentration of 25-OHD_3_ by NIST: 18.07 ng/mL.

^a^Single step extraction.

^b^Re-extraction of pellet.

^c^Two-step extraction.

**Table S2. Recovery experiment performed by standard addition of [^2^H_6_]25-OHD_3_ to human serum.**

| Amount added (ng) | Experimental ratio  25-OHD_3_/[^2^H_6_]25-OHD_3_ | Theoretical ratio  25-OHD_3_/[^2^H_6_]25-OHD_3_ | % Recovery^a^ | Mean % recovery | CV  (%) |
| --- | --- | --- | --- | --- | --- |
| [^2^H_6_]25-OHD_3_ | | | | | |
| *1* | 4.94 |  | | | |
|  | 4.93 |  |  |  |  |
|  | 5.10 |  |  |  |  |
| *2* | 2.59 | 2.47 | 104.7 | 102.0 | 3.6 |
|  | 2.55 | 2.46 | 103.6 |  |  |
|  | 2.49 | 2.55 | 97.8 |  |  |
| *4* | 1.32 | 1.24 | 107.2 | 106.3 | 4.1 |
|  | 1.36 | 1.23 | 110.1 |  |  |
|  | 1.30 | 1.28 | 101.6 |  |  |
| *6* | 0.86 | 0.82 | 105.0 | 104.6 | 0.8 |
|  | 0.85 | 0.82 | 103.7 |  |  |
|  | 0.89 | 0.85 | 105.3 |  |  |

Serum used batch DEQAS423. Predetermined concentration of 25-OHD_3_ using the presented methodology: 29.45 ng/mL.

^a^ % Recovery = {([25-OHD_3_]/[^2^H_6_]25-OHD_3_)_exp_/([25-OHD_3_]/[^2^H_6_]25-OHD_3_)_theor_} x 100 %

**Table S3. Recovery experiment performed by standard addition of 25-OHD_3_ to human serum.**

| Amount added (ng) | Experimental concentration 25-OHD_3_ (ng/mL) | Theoretical concentration 25-OHD_3_ (ng/mL) | % Recovery^a^ | Mean % recovery | CV  (%) |
| --- | --- | --- | --- | --- | --- |
| 25-OHD_3_ | | | | | |
| *0* | 16.54 |  | | | |
|  | 16.05 |  |  |  |  |
|  | 15.99 |  |  |  |  |
| *1* | 26.49 | 26.54 | 99.5 | 104.9 | 4.5 |
|  | 26.78 | 26.05 | 107.3 |  |  |
|  | 26.77 | 25.99 | 107.8 |  |  |
| *2* | 35.62 | 36.54 | 95.4 | 101.2 | 5.3 |
|  | 37.25 | 36.05 | 106.0 |  |  |
|  | 36.45 | 35.99 | 102.3 |  |  |
| *4* | 59.37 | 56.54 | 107.1 | 104.1 | 3.7 |
|  | 58.19 | 56.05 | 105.4 |  |  |
|  | 55.89 | 55.99 | 99.7 |  |  |
| *6* | 76.42 | 76.54 | 99.8 | 101.8 | 2.2 |
|  | 78.57 | 76.05 | 104.2 |  |  |
|  | 76.89 | 75.99 | 101.5 |  |  |

Serum used batch DEQAS424.

^a^ % Recovery = {[25-OHD_3_]_exp_/[25-OHD_3_]_theor_} x 100 %
